# Goal formulation and tracking in child mental health settings: when is it more likely and is it associated with satisfaction with care?

**DOI:** 10.1007/s00787-016-0938-y

**Published:** 2017-01-18

**Authors:** Jenna Jacob, Davide De Francesco, Jessica Deighton, Duncan Law, Miranda Wolpert, Julian Edbrooke-Childs

**Affiliations:** 1 Child Outcomes Research Consortium, Evidence Based Practice Unit, University College London and the Anna Freud Centre, 21 Maresfield Gardens, NW3 5SD London, UK; 2 Evidence Based Practice Unit, University College London and the Anna Freud Centre, London, UK; 3 London and South East CYP-IAPT Learning Collaborative, Hosted by the Anna Freud Centre, London, UK

**Keywords:** Collaborative practice, Goals, GBOs, Satisfaction, CAMHS, Shared decision-making

## Abstract

Goal formulation and tracking may support preference-based care. Little is known about the likelihood of goal formulation and tracking and associations with care satisfaction. Logistic and Poisson stepwise regressions were performed on clinical data for *N* = 3757 children from 32 services in the UK (*M*_age_ = 11; SD_age_ = 3.75; most common clinician-reported presenting problem was emotional problems = 55.6%). Regarding the likelihood of goal formulation, it was more likely for pre-schoolers, those with learning difficulties or those with both hyperactivity disorder and conduct disorder. Regarding the association between goal formulation and tracking and satisfaction with care, parents of children with goals information were more likely to report complete satisfaction by scoring at the maximum of the scale. Findings of the present research suggest that goal formulation and tracking may be an important part of patient satisfaction with care. Clinicians should be encouraged to consider goal formulation and tracking when it is clinically meaningful as a means of promoting collaborative practice.

## Introduction

**Fig. 1:**
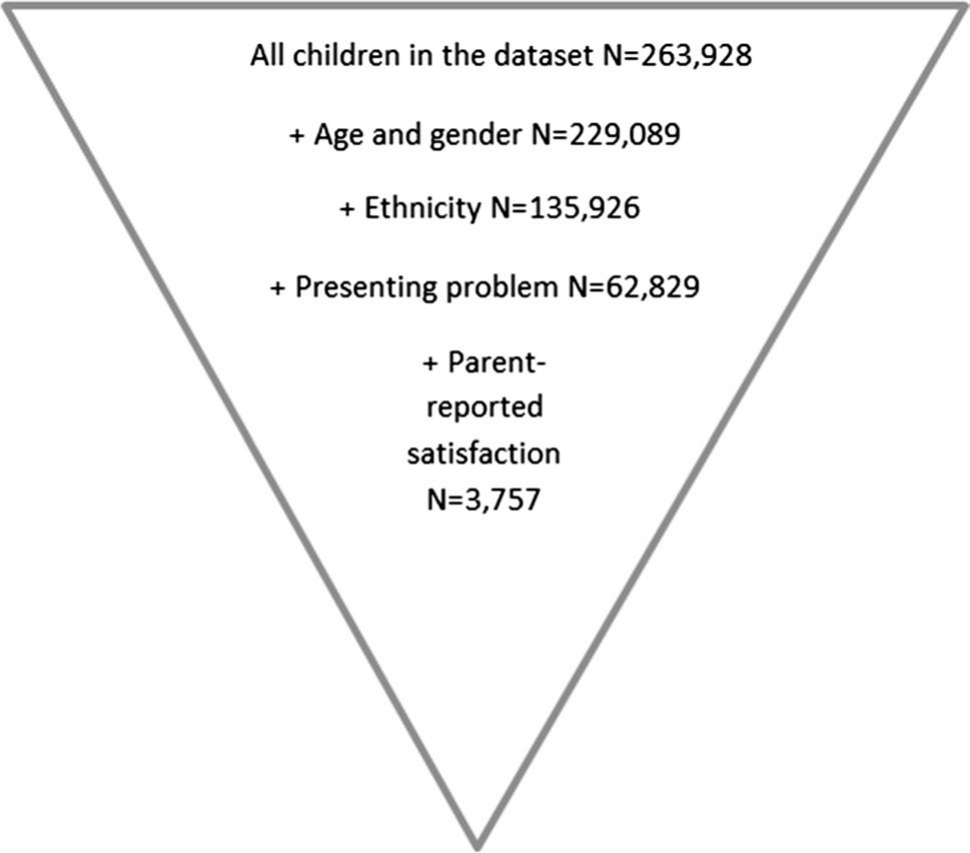
Sampling diagram

The use of goal formulation and tracking has been implemented in mental health settings in the United Kingdom (UK) and North America for some time (e.g. Hopes and Expectations [69]; Goal Attainment Scaling (GAS) [46]; Top Problems [9]) and is seen as an important part of many evidence-based practices (e.g. [7, 17, 32]). However, other than general life goals (e.g. school attendance) set in accordance with personal characteristics (e.g. previous experiences, presenting difficulties and developmental stages; [23, 56, 57]), very little is known about the use of this approach with children in therapy. This includes what characteristics (including demographics such as age, gender, ethnicity or presenting problem) (Fig. 1) may determine the propensity to formulate goals and track goal progress and the potential impact doing so might have on experience of care received. In the present article, goals are specific outcomes a child, young person, or family wants to achieve in accessing mental health services [48]. Commonly set goals by children accessing services include coping with specific difficulties, personal growth, and independence, and commonly set goals by parents accessing services include managing specific difficulties their child has, parent-specific goals such as increased knowledge of their child’s difficulties, and improving self or life [44]. It has been suggested that goal formulation and tracking may be especially useful for service users with particular needs, where progress may not be expected in terms of symptom reduction [6], which may include children with learning disabilities or developmental difficulties.

There are a number of barriers to formulating and tracking goals in child mental health practice, including the child’s capacity to do so [29, 64]; involvement of parents who may have different experiences and perceptions to the child or their own emotional and behavioural difficulties [75]; lack of resources to record goals formally (or the pressure to do so) and training on setting and tracking meaningful goals [34, 61]; and safeguarding concerns around balancing the involvement of the child with their vulnerability [2, 71]. Some models of therapy (e.g. solution-focused therapy; [66]) use the ‘miracle question’ as a means to conceptualise what would be different if one’s difficulties magically disappeared. The ‘miracle question’ can be one way of helping to formulate goals [48]. However, there is no governance about how treatment goals should be set and tracked in child mental health practice. This means that very little is known about how practitioners help to formulate and track goals with children.

Adults accessing mental health support have been found to highly value working towards their own goals [50]. Parents of children attending UK child and adolescent mental health services (CAMHS) have advocated for more collaborative methods of outcome monitoring [54]. Practitioners and children report reviewing goals and tracking them as helpful to assisting communication, motivating children, and giving them ownership of their care [61]. Evidence also suggests that agreement amongst families and with practitioners about desired outcomes may in turn be associated with better outcomes [52] and reduced attrition [14, 70]. However, goals are still not widely routinely recorded and tracked, as demonstrated through the high proportion of missing data of this kind in routinely collected datasets (e.g. [73]).

Still, therapeutic goal setting in general is argued to be a way to focus all parties to work together towards reaching common, concrete and measureable objectives. Practitioners have also suggested that it is useful to track outcomes such as goal progress over time in order to monitor treatment progress [6, 61]. Setting and tracking goals with families has also been found to be a valuable way to gather further clinical information [27] and an explicit focus on patients’ goals has been identified as key to effective care [30]. An assessment process that incorporates goal formulation and tracking has been shown to help the practitioner to understand the patient’s difficulties in more depth and may also lead to positive alliance with the practitioner [3]. This kind of collaborative working is likely to affect the way parents and young people view and experience the care provided in terms of feeling listened to, feeling supported and feeling like the care addressed the issue they were concerned about. The impact of goal setting and tracking on experience of care has not yet been explored in detail; therefore, it has been chosen as a focus for the present research.

Experience of care is also a key dimension of healthcare quality. Groups of practitioners offering provision to children, young people and their families with mental health and wellbeing difficulties are striving to improve experience by providing more person-centred care and tailoring delivery to an individual’s needs [22, 43]. Experience of care has been shown to have good face validity and may be better understood by professionals than more complex outcome information related to symptomology [33, 59]. Ratings of satisfaction are also sometimes the only proxy indicator available to assess the quality of a mental health intervention [8] which allows the service users’ voice to help inform an evaluation of support received [33].

Satisfaction with treatment has been demonstrated to be a complex factor, which may not have strong links to child-reported clinical outcomes [33] but may be associated with treatment adherence, perhaps due to its close links to shared decision-making. While links between outcome and satisfaction have not been found from a child’s perspective, they have been found from a parent’s perspective [10]. We are also interested in parental satisfaction in the present research due to the central role parents play in their child’s care, particularly in terms of engagement and adherence to treatment. Moreover, the therapeutic context is particularly complex as it involves a multidisciplinary, systemic approach with a number of stakeholders, perspectives and priorities. Evidence suggests that children, their parents, and therapists may have different perceptions of presenting problems and reasons for attending therapy [11, 40, 45]. Parents may set different goals to children, [35, 39] such as the management of specific symptoms, behaviour management, improving self-care and goals for parents themselves to achieve [44], which raises the important question of who is the patient: the child or the parent?

Parental investment in and agreement of goals may be crucial. Parental satisfaction has been indicated to be a key part of therapy completion in child mental health [5] due to the integral role parents often take in the management of a child’s emotional and behavioural difficulties [62]. A parent’s experiences of the support received and their relationship with the practitioner have been shown to be associated with parental satisfaction with child mental health care [42, 62].

Further, much of the work done related to child mental health is family-centred work, e.g. systemic family therapy in the UK and family systems interventions in America [24, 53], and there has been a call to explore other types of outcome measures for use in this work [41]. Evidence has shown that aside from demographic characteristics, the majority of variance in parent- and child-rated satisfaction of mental health treatment remains unexplained. As such, satisfaction may be influenced by factors that are relatively unexplored, such as ‘customer relations’-type variables, therapeutic alliance (e.g. a positive association has been found between parental satisfaction and length of treatment [13, 36, 49]) or physical characteristics of the experience [33] which could be due to service-level variance, i.e. that there are things specific to the service visited or received that have an impact on satisfaction.

Goal setting and tracking may promote collaborative practice by enhancing child and parental communication with practitioners [61]. In good working relationships, patients and practitioners work together to agree goals, discuss options for preference-based care and agree ways forward [12, 16, 20, 27, 55, 61, 65], which is also a predictor of positive treatment outcome [9, 40, 68].

The above evidence suggests that goal formulation during therapy may be associated with a better experience of support received, as it might promote communication and collaborative practice between practitioners and children, young people and parents, and therefore more positive experiences of and satisfaction with care. To the best of our knowledge, there is no existing evidence on whether goal formulation and tracking is more likely in therapeutic work with certain children and young people, in terms of demographic and case characteristics, or on whether goal formulation is associated with satisfaction with care received.

## Aims of the present research

The aim of the present research was to explore whether goal formulation and tracking was more likely in therapeutic work with children and young people with certain demographic (i.e. age, gender, ethnicity) and case characteristics (i.e. presenting problems). We also sought to explore the association between goal formulation and tracking in therapeutic work with children and young people during therapy, and carer-reported satisfaction with care received.

## Method

### Participants and procedure

Data from children and young people attending child mental health services, including characteristics about the child or case and their outcome information, were submitted to the Child Outcomes Research Consortium (CORC; [28]) as part of routine outcome monitoring practice. At the time of the research, the main dataset constituted 263,928 cases, from 1990 until 2013. Services include a range of inpatient and outpatient service provided by the State and also by voluntary sector organisations. Not all patients seen by services were included in the dataset as only a subset of data are shared with the CORC team, for various reasons, including barriers to collecting the data, which have been discussed elsewhere (e.g. [28, 72]). The children, young people and their families seeking support receive support for a range of mental health and wellbeing difficulties, including emotional, behavioural and developmental difficulties. The type of support offered varies and includes services such as counselling, cognitive behaviour therapy (CBT), parent training and art therapies; mainly provided by multidisciplinary teams.

The data were filtered so that cases were included that met the following criteria: children seen for support during the time of data collection 2007 and 2013 (from when the bulk of support providers began to submit substantial datasets until the time of analysis); gender, age, ethnicity, at least one presenting problem, and parent-reported satisfaction with service were present (i.e. not missing and not ‘not known’). This resulted in a sample of *N* = 3757 children from 32 child mental health services across the UK. The majority of the services were all outpatient clinics and part of the National Health Service with four services being voluntary sector providers of care.

The demographic characteristics and problem types of the total sample, those with goals present, and those with goals absent are shown in Table 1. Overall, 54% of children were male, which is consistent with findings from national surveys of children’s mental health in the UK [38]. The mean age was 11 years (SD = 3.8), and the most common types of clinician-recorded presenting problems were emotional difficulties (55.6%), followed by ‘other’ difficulties (20.7%), and behavioural difficulties (15.1%) (see Table 1); national surveys in the UK have reported behavioural difficulties as the most common problem among children, followed by emotional difficulties [38].

**Table 1:** Demographic characteristics and problem types

	Total sample	Goals present	Goals absent
*N*	3757	278	3479
Male, *n* (%)	2038 (54%)	170 (61%)	1868 (54%)
Age, Mean (SD)	11 (3.75)	8.96 (5.10)	11.16 (3.57)
Ethnicity, *n* (%)			
White	2582 (69%)	172 (62%)	2410 (69%)
Mixed	184 (5%)	22 (8%)	162 (5%)
Asian	156 (4%)	9 (3%)	147 (4%)
Black	159 (4%)	19 (7%)	140 (4%)
Other	163 (4%)	12 (4%)	151 (4%)
Not stated or missing	513 (14%)	44 (16%)	469 (14%)
Hyperactivity, *n* (%)	445 (12%)	22 (8%)	423 (12%)
Emotional problems, *n* (%)	2088 (56%)	170 (61%)	1918 (55%)
Conduct problems, *n* (%)	568 (15%)	37 (13%)	531 (15%)
Eating disorder, *n* (%)	201 (5%)	8 (3%)	193 (6%)
Psychosis, *n* (%)	41 (1%)	6 (2%)	35 (1%)
Self-harm, *n* (%)	248 (7%)	21 (8%)	227 (7%)
Autism, *n* (%)	409 (11%)	41 (15%)	368 (11%)
Learning disability, *n* (%)	236 (6%)	74 (27%)	162 (5%)
Developmental difficulties, *n* (%)	145 (4%)	19 (7%)	126 (4%)
Habit disorder, *n* (%)	136 (4%)	5 (2%)	131 (4%)
Substance abuse, *n* (%)	32 (1%)	3 (1%)	29 (1%)
Other problems, *n* (%)	777 (21%)	55 (20%)	722 (21%)

Presenting problems are not mutually exclusive

The most frequent ethnicity was White British (63.7%), followed by Any Other White Background (e.g. White European; 4.6%). This also fits with other research suggesting an under-representation of Black and Minority Ethnic (BAME) groups in CAMHS [51].

Unfortunately no practitioner-level data were available, a point that will be discussed in the limitations section. Each site had between 1 and 578 children per service. The most commonly assigned type of support received was indicated as ‘other’ type of therapy (19.1%), followed by CBT (13.2%) and family therapy (6.9%) (not exhaustive).

## Measures

### Goal formulation present vs. absent

To measure the presence vs. absence of goal formulation, the Goal-based Outcomes (GBOs) tool was used [47]. The GBOs tool is a measure of progress towards achieving goals for therapy, which are set by practitioners, children, parents, and ideally mutually agreed. A total of three goals can be formulated, with progress towards each reported on an 11-point scale from 0 (“no progress has been made”) to 10 (“goal has been fully reached”). Unlike standardised patient-reported outcome measures (PROMs), goal setting can be uniquely tailored to each child and may capture outcomes in areas that are not explored by other measures, such as broad asset-based dimensions including self-confidence, resilience and self-esteem [44].

The GBOs is a widely used goals tool in child mental health settings across the UK (GBO; [47]; see also [19]). Significant correlations with other clinician-, parent- and child-rated standardised measures (*r* between 0.10 and 0.39) have been evidenced [73]. The internal consistency has also been found to be acceptable, suggesting that despite criticisms about a lack of comparability due to diverse goals [63], GBO may in fact be measuring the same underlying construct. Goals have also been demonstrated as showing more change over time than psychosocial difficulties and the impact of difficulties on the child’s life. The relationship between change in goals with functioning and perceived satisfaction were also stronger than the relationships with change in symptomology or the impact on everyday life [25].

Goals are set and recorded at the start of therapy where baseline level of progress is recorded, to be tracked. Detailed guidance is available on goal setting and tracking, and it is recommended that goals are specific, measurable, attainable, realistic, and timely; problem focussed; and collaborative [48]. In particular, guidance highlights that the aims of a goal should be to inform direction of therapeutic work and should be achievable but also challenging. A dichotomous variable was created for the present research to distinguish children with goal formulation and tracking present (a baseline progress towards goals score of 0–10) from goal formulation and tracking absent (a missing baseline progress towards goals score). This resulted in 278 cases with goal formulation present and 3479 with goal formulation absent. Initial analyses showed a substantial variation in presence of goal formulation and tracking between services (the intraclass correlation coefficient was 0.39), suggesting that a multilevel analysis was appropriate.

### Satisfaction with care (parent-reported)

To measure satisfaction with care received, the 9-item “satisfaction with care” subscale of the Experience of Service Questionnaire (ESQ) [5] was used (also see [15]). The ESQ is a 12-item measure of experience of support received, capturing perceived satisfaction with care [nine items; e.g. “I feel that the people who saw me listened to me”) and satisfaction with the environment (three items; e.g. “The facilities here are comfortable (e.g. waiting area)”].

The ESQ is completed approximately 6 months after the start of treatment or at case closure (discharge) if that is sooner. Parents responded to items on a 3-point scale from 0 (“certainly true”) to 2 (“not true”) and these scores are summed to obtain an ‘overall satisfaction with care’ score ranging from 0 (completely satisfied) to 18 (completely unsatisfied). There are three additional items that are not used to compute subscales as they require open-ended responses, the data from which were not analysed in the present research. The ESQ has been used in previous research, in which it demonstrated acceptability and internal consistency (Cronbach’s alpha = 0.88; [25]). In the present research, the Cronbach’s alpha was 0.86. A small between-service variation of the satisfaction with care (intraclass correlation coefficient was 0.10) was found; therefore, the multilevel approach was not considered necessary for this analysis.

## Analytic strategy

To explore the relationships between demographic characteristics, presence vs. absence of goal formulation and tracking, and satisfaction with care, two sets of analyses were performed.

First, to explore differences between children with goal formulation and tracking present and children with goal formulation and tracking absent, a multilevel (with children clustered within services) logistic regression predicting the presence vs. absence of goals with the demographic characteristics (age, gender, ethnicity, presenting problems) was conducted. Here, a stepwise model selection approach was used, meaning predictors were only retained in the model if they improved the model fit. This means that all demographic and case characteristics (including the presence of possible combinations of presenting problems recorded for at least 20 children; e.g. hyperactivity × conduct difficulties present vs. absent) were initially entered in the model but were only retained and reported in the Results section if they improved the model fit.

Second, to explore differences between children of parents who were completely satisfied with care and children of parents who were not completely satisfied with care, a zero-inflated Poisson regression predicting parents’ satisfaction scores with demographic characteristics and goal formulation and tracking present vs. absent was conducted. This regression model has two parts: a logistic model for predicting parents being completely satisfied with care (scoring 0) vs. not being completely satisfied with care (scoring 1–18) and a Poisson count regression predicting the actual satisfaction with care score. The choice of this model was motivated by the high number of parents being completely satisfied with care [2415 of 3757 (64.5%)] and by the distribution of scores greater than zero.

This dovetails with previous research, showing that service users are likely to report extremely high levels of satisfaction with support received [15]. In both regressions, stepwise model selection was used, whereby predictors were only retained when their inclusion improved the overall model fit.

Listwise deletion of parents’ satisfaction with care was used rather than substitution (e.g. with mean scores). Attrition in mental health settings has been shown to be particularly high, and CAMHS is no exception, with up to 40% of children and parents not completing treatment [58]. Attrition in child mental health services is complex and the reasons children and their parents may stop attending services are multi-faceted [4]. Parents who are less satisfied may be more likely to stop attending and complete measures [5]; therefore, mean substitution did not seem appropriate.

## Results

Table 2 shows the results of the multilevel logistic regression for demographic characteristics predicting the presence vs. absence of goal formulation and tracking. Table 3 shows the results of the presence vs. absence of goals predicting parents being completely satisfied with care vs. not being completely satisfied with care, and also their satisfaction with care score (on the logarithm scale), after controlling for demographic characteristics.

**Table 2:** Logistic regressions for demographics characteristics predicting presence vs. absence of goal formulation

Predictor	*β*	SE	*P*	OR (95% CI)
Intercept	–6.49	1.37	<0.01	
Aged 6–12 vs. 0–5	–0.50	0.25	0.05	0.61 (0.37, 0.99)
Aged 13–18 vs. 0–5	–0.23	0.28	0.41	0.79 (0.46, 1.38)
Autism present vs. absent	–0.24	0.38	0.52	0.78 (0.37, 1.66)
Hyperactivity present vs. absent	–0.44	0.44	0.31	0.64 (0.27, 1.53)
Conduct problems present vs. absent	–0.52	0.33	0.12	0.59 (0.31, 1.14)
Self-harm present vs. absent	–1.11	0.85	0.20	0.33 (0.06, 1.74)
Learning disability present vs. absent	2.10	0.28	<0.01	8.13 (4.72, 14.14)
Hyperactivity × conduct difficulties present vs. absent	2.60	0.75	<0.01	13.44 (3.10, 58.56)
Emotional problems × self-harm present vs. absent	1.58	0.92	0.09	4.84 (0.80, 29.46)
Autism × learning disability present vs. absent	1.00	0.60	0.10	2.72 (0.84, 8.81)

*N* = 3757. A stepwise model selection was used, meaning predictors were only retained in the model if they improved the model fit

*SE* standard error, *OR (95% CI)* odds ratio (with 95% confidence interval)

Service-level random effects variance (standard deviation): 24.8 (4.98)

**Table 3:** Zero-inflated Poisson regression of presence vs. absence of goal formulation predicting parents’ satisfaction with care, controlling for demographic and case characteristics

Probability of parents being completely satisfied (scoring 0 vs. scoring 1–18)				
Predictor	*β*	SE	*P*	OR (95% CI)
Intercept	0.54	0.05	<0.01	
Goal formulation present vs. absent	0.68	0.16	<0.01	1.97 (1.44, 2.70)
Conduct problems present vs. absent	–0.16	0.10	0.09	0.85 (0.70, 1.04)
Self-harm problems present vs. absent	–0.30	0.14	0.03	0.74 (0.56, 0.97)
Autism problems present vs. absent	–0.31	0.12	0.01	0.73 (0.58, 0.93)
Learning disability problems present vs. absent	–0.32	0.18	0.07	0.73 (0.51, 1.03)
Other problems present vs. absent	0.16	0.09	0.07	1.18 (0.98, 1.40)
Autism × learning disability present vs. absent	0.86	0.37	0.02	2.37 (1.14, 4.88)

Parents’ satisfaction with care score (on the logarithm scale)				
Predictor	*β*	SE	*p*	D (95% CI)
Intercept	1.12	0.04	<0.01	
Goal formulation present vs. absent	–0.40	0.10	<0.01	–1.00 (–1.38, –0.57)
Ethnicity: mixed vs. White British	–0.06	0.08	0.42	–0.18 (–0.60, 0.31)
Ethnicity: Asian vs. White British	–0.22	0.09	0.02	–0.59 (–1.00, –0.13)
Ethnicity: Black vs. White British	–0.40	0.09	<0.01	–1.01 (–1.34, –0.61)
Ethnicity: other vs. White British	–0.26	0.09	<0.01	–0.71 (–1.08, –0.25)
Ethnicity: not stated vs. White British	–0.05	0.05	0.28	–0.15 (–0.42, 0.15)
Emotional problems present vs. absent	0.16	0.04	<0.01	0.54 (0.26, 0.83)
Hyperactivity problems present vs. absent	0.14	0.05	<0.01	0.46 (0.13, 0.82)
Conduct problems present vs. absent	0.26	0.04	<0.01	0.92 (0.61, 1.23)
Self-harm problems present vs. absent	0.27	0.05	<0.01	0.94 (0.58, 1.36)
Autism problems present vs. absent	0.17	0.05	<0.01	0.56 (0.23, 0.94)
Learning disability problems present vs. absent	0.06	0.08	0.43	0.20 (–0.28, 0.74)
Habit problems present vs. absent	–0.65	0.13	<0.01	–1.45 (–1.82, –1.00)
Other problems present vs. absent	0.08	0.06	0.14	0.27 (–0.11, 0.67)
Autism × learning disability present vs. absent	–0.41	0.20	0.04	–1.03 (–1.69, –0.05)
Hyperactivity problems × conduct problems present vs. absent	–0.30	0.11	<0.01	–0.78 (–1.23, –0.25)
Emotional problems × other problems present vs. absent	–0.24	0.09	<0.01	–0.65 (–1.04, –0.19)

*SE* standard error; *OR* odds ratio, *D (95% CI)* difference on the natural scale (with 95% confidence interval)

*N* = 3757

“Habit” problems refer to compulsions such as handwashing, hair pulling and includes obsessive compulsive disorder. “Other” problems refer to any other problems not indicated in the available list of variables

The first aim of the present research was to explore whether goal formulation and tracking was more likely in therapeutic work with children with certain demographic (i.e. age, gender, ethnicity) and case characteristics (i.e. presenting problems). Regarding demographic characteristics, children aged 6–12 were less likely (OR = 0.61, *p* = 0.05) to have goal formulation and tracking present than children aged 0–5. Regarding presenting problems, children with learning disability were more likely (OR = 8.13, *p* < 0.01) to have goal formulation and tracking present than children without learning disability.

The presence of both hyperactivity × conduct difficulties was also significant, and children with both these presenting problems were more likely (OR = 13.44, *p* < 0.01) to have goal formulation and tracking present than children without one of these presenting problems. The standard deviation of service-level random effects was 4.98 suggesting the between-service variation in the probability to have goal formulation and tracking was large, even after differences in age and presenting problems were taken into account.

The second aim of the present research was to explore the association between goal formulation and recording in therapeutic work with children during therapy and satisfaction with care received. Parents of children with goal formulation present reported on average one point (on the log-scale *β* = –0.4, *p* < 0.01) higher in satisfaction with care compared to parents of children without goal formulation, even after controlling for demographic and case characteristics.

## Discussion

The aim of the present research was to explore whether goal formulation and tracking was more likely in therapeutic work with children with certain demographic and case characteristics. We also sought to explore the association between the presence of goal formulation and tracking in therapeutic work with children during therapy and satisfaction with care received. Routinely collected data from children attending child mental health services in the UK were analysed.

Pre-school children (0–5 years) were marginally significantly more likely to have goals formulated and tracked than older children, which may reflect goal setting and tracking filling a gap in standardised PROMs as there are few other tools available to measure change in this age group. While standardised measures do potentially measure different constructs to goals, self-report measures for young children are sparse due to the cognitive abilities of this age group; this can be evidenced by their scant presence in the Children and Young People’s Improving Access to Psychological Therapies (CYP IAPT) programme [21], a UK government-funded initiative seeking to transform CAMHS through routine outcome monitoring. Standardised self-report measures such as the Strengths and Difficulties Questionnaire [37] often have a lower age limit of 11 and thus parent reports are relied upon.

Possibly for similar reasons, children with learning disabilities were more likely to have goals formulated and tracked. Children in this group may be subject to similar challenges to younger children, in that the completion of standardised measures requires a certain level of literacy. This may in part explain the association between children with learning disabilities with presence of goal formulation and tracking. Alternatively, it may suggest that practitioners feel more structure is required with these populations and therefore actively choose to work on goals. It could also support the suggestion that goal formulation and tracking is helpful with children with whom standardised measures may be less applicable or less likely to capture changes in symptoms and functioning [6].

Children presenting with both hyperactivity and conduct disorder were more likely to have goals formulated than children without these presenting problems, and with these presenting problems alone. Children seen for hyperactivity along with conduct difficulties may have been seen for ongoing psychological intervention, meaning formulation and tracking of goals may have been more likely. This finding may also be due to practitioners providing additional intensive interventions to meet the needs of these children and their parents. For example, there may be differences in services providing care for children with multiple diagnoses to those seeing children with fewer diagnoses or different types of complexities.

There were large differences between services even when allowing for these factors. This suggests that there may be differences in organisational culture and training in relation to goal setting (and potentially collaborative practice) that may be of relevance. It is known that the move to more collaborative ways of working is novel for some practitioners and there are concerns about this approach (see [25]). In particular, the fact that the majority of cases did not have any evidence of goals being formulated (98.5% of the overall dataset) suggests that this practice has yet to find widespread adoption. There are promising indications that training practitioners to become more collaborative in their work with children can improve their skills and motivation [26]. The current results suggest more targeted training for practitioners working with key groups, particularly those not being currently worked with in terms of goal formulation, may also be warranted.

There may of course be other reasons for the high proportion of cases where goals were not set and tracked, including a lack of resources to record goals formally and a reliance on the subjective views of children [61]. More generally, practitioners may have concerns that outcome measurement of this kind does not capture case complexity, will increase burden or will be misinterpreted [74]. Others have reported barriers from children themselves, related to not feeling that they have the capacity to set goals [67].

Moreover, there may be overarching service-level reasons for not using a particular form of outcome measurement. For example, a commissioning body might make funding decisions based on specific measures, which are often more generalisable and normed measures. Alternatively, practitioners may not feel as though goal setting and tracking fits with their client group; for example, it is unlikely that goals would be set for a child attending a service every 6 months for a medication review.

The findings of the present research suggest that formulating and tracking goals may be associated with higher levels of parental satisfaction, as parents were much more likely to be completely satisfied with care and, on average, rated satisfaction one point higher when goals were formulated and tracked; however, this does not suggest causation and the meaning of a one-point difference in satisfaction is not clear and perhaps likely to be small. What is less clear is what might contribute to this effect. It may be that the mere act of goal formulation communicates a message of collaboration to families that increases satisfaction with the support received. However, it is likely that there are more active ingredients at play: due to the variance between services found, the absence or presence of goal formulation in the dataset may be a reflection of a more or less positive culture of individual therapists or teams. The helpful clinical processes that go on around goal formulation are not yet understood, and further research is needed to understand the ‘active ingredients’ that contribute to effective goal formulation.

Although overall rates of satisfaction were already high, this sample may be positively skewed due to differential drop-out, and the increased satisfaction of those with goals may be even higher in the sample not captured. This finding may suggest that goal formulation and tracking may be associated with higher levels of parental satisfaction, potentially through higher levels of parental involvement and collaborative working, as suggested by prior research [2, 42], and may highlight the association between therapeutic relationships and the importance of goal formulation and tracking [12, 16, 20, 27, 55, 61, 65]. It may of course also be possible that this association is more to do with another common factor such as greater therapeutic alliance to which both higher levels of goals and satisfaction relate, but given the existing literature on how goal setting might help support this it is not unreasonable to at least consider goal setting as enhancing satisfaction and collaborative working.

The finding that parents of children with self-harm were less likely to be completely satisfied with care may be an artefact of the intrinsic nature of those difficulties; for example, parental ambivalence towards receiving treatment for their child’s self-harm has been discussed elsewhere, which may in turn affect their perception of satisfaction of this care [60]. However, there may be other factors related to treatment received, individual differences in patients and practitioners (e.g. ethnicity as also found here), or organisational culture that influenced the findings.

### Limitations

Limitations should be considered when interpreting the findings of the present research. First, there is much to consider in terms of how goal setting is presented to children. For example, one may presume that goal formulation makes parents feel as though they are all working together cohesively, which should be investigated in future research. Similarly, we were unable to explore whether who formulated and tracked goals was associated with satisfaction with care received. Future research should explore whether goal formulation and tracking shows different associations with satisfaction with care depending on who formulates and tracks goals and who rates satisfaction with care. Linked to this, it was not possible to ascertain how far the goal formulating and tracking was a collaborative process, which means the relationship found between the presence of goals and satisfaction cannot easily be attributed to increased engagement or shared working, but is suggested based on learning from prior research. In particular, goal formulation and tracking was more likely with younger children perhaps because it was easier for parents to agree about treatment goals than with older children.

Second, attrition in mental health settings has been shown to be particularly high. In some areas of the UK, as many as 40% of those seen by mental health services have been found to disengage for a variety of reasons, including family disadvantage [58]. Because naturalistic research is a measure of everyday life, participants may not have the commitment they might have had to providing data in a randomised control trial. Moreover, the practice of goal formulation can feel strange to practitioners and has, as part of collaborative practice, been described as feeling ‘clunky’ [2] perhaps especially when the approach has not been implemented in a well-considered manner, such as having open discussions and training [67]. Parents who are less satisfied may be more likely to disengage and not provide outcome or feedback information [5]. Goals may have been formulated but not captured in the dataset. The data included in this research were a subset of a wider dataset, from a self-selecting range of services, leading to unknown representativeness. Some reasons for the high proportion of cases without goals have been discussed. Missing data creates ambiguity which may be because systematic biases are likely in those who do provide their data [18]. Future research should examine whether there is an association between goal formulation and tracking and attrition.

Third, because the most common therapy type was indicated as ‘other’ (19%) it limits the ability to understand the impact of our findings across a range of interventions and the generalisability of these findings. Although diagnostic criteria are not routinely used in child mental health services in the UK, more detailed information on young people’s presenting problems may have enabled us to tease out effects about for whom goal formulation and tracking is most associated with satisfaction with care. Similarly, without a randomised control design, inferences of causation should not be made and it is possible that differences in unobserved characteristics between the two groups, which were also of different sizes, explain the pattern of associations.

Fourth, as already noted above, we did not have data to allow us to consider the data as nested in individual practitioners as well as services, and there were not sufficient available data to examine these associations between practitioner and organisational characteristics, and practitioner behaviour and satisfaction with care; however, we are currently addressing this limitation in an in-progress study examining the association between organisational social context and practitioner attitudes in child mental health services (also see [1]).

Finally, a cross-sectional measure of satisfaction with care may not adequately capture a transient phenomenon. Emerging studies suggest that change in therapeutic alliance is the key factor, as opposed to a static measurement of satisfaction [9]. This suggests that practitioners should be capturing this information on a regular basis and using it to inform their own practice and work with children and families.

### Recommendations for practice and concluding comments

Goal formulation and tracking early on in the child or family’s contact with support providers can help make better, collaborative choices about treatment options; goal clarity can help facilitate better choice of interventions that fit the needs and wishes of the patient, leading to better clinical outcomes [31] and better experience of support received. Approaches that have goal formulation and tracking as an integrated part of a family’s journey through mental health and counselling services, such as the Choice and Partnership Approach (CAPA; [76]), see collaborative goal setting as vital components in developing joint formulations of a presenting difficulty, and allow the discussion of treatment options: ‘choice’ would seem to be supported by these findings.

The findings of the present research also suggest that practitioners may choose to formulate and track goals with certain children and parents based on individual characteristics, including particular presenting difficulties and other characteristics, which should be further investigated in future research, particularly in light of the associations between goal formulation and tracking and satisfaction with care. There may be several factors which affect a practitioner and child’s decision to use or not use goals in their work together and some of these have been suggested. One barrier that does arise in previous research is the need for better training in the use of goal formulation and tracking in these settings; given the high proportion of cases without goals, as well as the inter-service differences, this is a recommendation of this research. Targeted training in this area for practitioners working with key groups may be particularly warranted due to the positive effects shown as a result of such training in previous research [26].

The research here and elsewhere suggests that goal formulation and tracking may be more widely used with children with particular characteristics, which may be because other outcome tools leave a gap due to, for example, the age or presenting problem of the child, or because they capture other aspects of care not covered by standardised measures; e.g. coping and resilience [44]. It would also be useful to further explore whether goals were routinely used alongside or instead of other measures. For example, the finding that parents of children with goal formulation present were more satisfied with care than those without could be related to the use of any measure rather than specifically related to the use of goals.

The findings of the present research suggest that goal formulation and tracking may be associated with higher levels of parental satisfaction, which supports prior research indicating that goals—as part of collaborative practice—can focus treatment and support more preference-based care [3, 55]. In turn, this may have a positive impact on parental satisfaction [42] which strengthens the argument for further support to practitioners in goal formulation and tracking.

## Appendix

Goal-based Outcomes (GBOs) tool.

ESQ parent version.
